# Nature-Inspired Surface Structures Design for Antimicrobial Applications

**DOI:** 10.3390/ijms24021348

**Published:** 2023-01-10

**Authors:** Meng-Shiue Lee, Hussein Reda Hussein, Sheng-Wen Chang, Chia-Yu Chang, Yi-Ying Lin, Yueh Chien, Yi-Ping Yang, Lik-Voon Kiew, Ching-Yun Chen, Shih-Hwa Chiou, Chia-Ching Chang

**Affiliations:** 1 Department of Medical Research, Taipei Veterans General Hospital, Taipei 112201, Taiwan; 2 Institute of Pharmacology, National Yang Ming Chiao Tung University, Taipei 112304, Taiwan; 3 Department of Biological Science and Technology, National Yang Ming Chiao Tung University, Hsinchu 300193, Taiwan; 4 Department of Botany and Microbiology, Faculty of Science, Al-Azhar University, Assiut Branch 71524, Egypt; 5 Department of Biomedical Sciences & Engineering, National Central University, Taoyuan City 320317, Taiwan; 6 Department of French Language and Literature, National Central University, Taoyuan City 320317, Taiwan; 7 Department of Pharmacology, Faculty of Medicine, Universiti Malaya, Kuala Lumpur 50603, Malaysia; 8 Department of Electrophysics, National Yang Ming Chiao Tung University, Hsinchu 300093, Taiwan; 9 Center for Intelligent Drug Systems and Smart Bio-devices (IDS2 B), National Yang Ming Chiao Tung University, Hsinchu 300193, Taiwan; 10 Institute of Physics, Academia Sinica, Nankang, Taipei 11529, Taiwan

**Keywords:** antimicrobial surface, anti-bacteria, anti-virus, anti-biofouling, structure, surface topography

## Abstract

Surface contamination by microorganisms such as viruses and bacteria may simultaneously aggravate the biofouling of surfaces and infection of wounds and promote cross-species transmission and the rapid evolution of microbes in emerging diseases. In addition, natural surface structures with unique anti-biofouling properties may be used as guide templates for the development of functional antimicrobial surfaces. Further, these structure-related antimicrobial surfaces can be categorized into microbicidal and anti-biofouling surfaces. This review introduces the recent advances in the development of microbicidal and anti-biofouling surfaces inspired by natural structures and discusses the related antimicrobial mechanisms, surface topography design, material application, manufacturing techniques, and antimicrobial efficiencies.

## 1. Introduction

Microbes are microscopic organisms that include bacteria, fungi, viruses, and specific eukaryotic species that are mostly found on Earth [1]. Cross-transmission by microbe-contaminated surfaces, such as bacterial biofilms on the surfaces of medical apparatus, may have severe consequences for patients [2]. Furthermore, the increased dose and duration of antimicrobial treatments may eventually induce the growth of antimicrobial-resistant species [3,4,5,6]. According to the World Health Organization (WHO), antimicrobial resistance (AMR) is one of the most significant threats to the current health system [7]. The WHO has estimated that AMR may cause the death of >10 million people annually by 2050 [8].

The surface contamination of tissues by viral particles is a critical health concern. For example, coronaviruses were found to remain viable on the surfaces of iron, cotton, and surgical masks from 10 h to >7 day [9], and severe acute respiratory syndrome coronavirus 2 (SARS-CoV-2), which causes COVID-19, was determined to be active for 72 h on soft plastics [10]. Although concrete proof has not been reported, there is a growing concern regarding the possible transmission of coronaviruses through contaminated surfaces [11]. Therefore, the development of novel, cost-effective, and non-labor-intensive surface decontamination strategies is highly desirable.

Currently, chemical sanitization and UV treatments are employed to decontaminate microbe-infected surfaces. However, repetitive chemical sanitization is not only labor-intensive and uneconomical but also causes environmental stress [12]. For example, chemical reagents used for sanitization, such as chlorine, hydrogen peroxide, sodium hypochlorite, and ethanol, may increase the probability of cardiorespiratory disease under long-term exposure to the human body [13] and cause skin irritation, dryness, and itching upon prolonged exposure [14]. UV treatment may cause skin burns and increase the risk of skin cancer [13].

In the past decade, the development and use of novel structure-related antimicrobial surfaces that actively prevent the surface adhesion and/or propagation of microbes have emerged as a sustainable and safe alternative to conventional approaches. They have increasingly attracted the interest of the research and healthcare communities.

**Figure 1:**
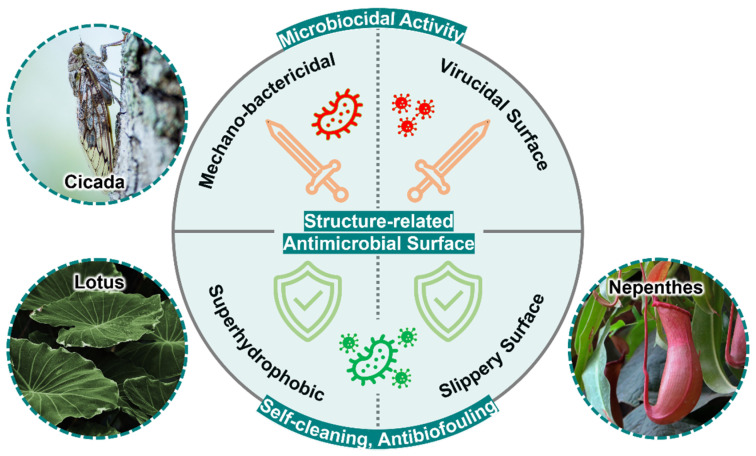
Classification of structure-related antimicrobial surfaces and their natural inspirations. The mechano-bactericidal surface and virucidal surface kill microbes; as superhydrophobic surfaces and slippery surfaces perform self-cleaning and anti-biofouling characteristics to prevent microbial adhesion. These surfaces are inspired by cicada wings, lotuses, and nepenthes, respectively.

The structure-related antimicrobial surfaces are categorized as microbicidal or anti-biofouling (Figure 1) [15]. In this review, we provide an update on the principles of microbial cell-surface interactions and discuss recent advances in the development of microbicidal and anti-biofouling surfaces. In addition, the related antimicrobial mechanisms, surface topography, material applications, manufacturing techniques, and pathogen tests are discussed.

## 2. Microbial Cell-Surface Interaction

The survival and reproduction of microbes on various surfaces depend on the physical absorption and/or chemical bond formation between the outer membrane of the microbes and the contact surface [16]. Therefore, understanding the interactions between the microbes and contact surfaces is vital for the development of novel antimicrobial surfaces.

Microbes are the oldest and largest organisms on earth, are distributed in various environments [17,18,19], and live within communities that play a vital role in the attachment and colonization of different surfaces. In addition, bacterial cells grow into planktonic and biofilm-forming cells. Bacteria in biofilms are relatively insensitive to antibacterial agents compared to their planktonic forms [20].

### 2.1. Bacterial Attachment and Colonization

Biofilms are formed by the attachment and colonization of microbes on immobilized surfaces [7,13,14] and occur in two phases. The first phase includes initial contact and attachment between the cell and immobilized surface by electrostatic and/or hydrodynamic interactions; this is a reversible process with a duration of up to 1 min [21,22]. The interactions between the cells and surface may be affected by the loss of interfacial water, structural modifications of the surface molecules, or modifications of the cell positions [23]. The second phase, the stabilization stage, involves van der Waals interactions between the hydrophobic region of the outer cell wall of the bacterial cell and the immobilized surface, lasting for up to several hours; this is an irreversible process [24]. Simultaneously, microbes produce extracellular polymeric substances (EPSs) to facilitate these interactions and protect colonies from environmental changes [2,8,13].

### 2.2. Extracellular Polymeric Substance

The extracellular matrix (ECM) in a biofilm is the most significant component of its formation and maintenance and comprises water and EPSs. The EPSs play a vital role in the interactions between bacterial cells and different surfaces; they are produced by microbes and include polysaccharides, proteins, and/or the DNA of the microbes [25,26]. Moreover, the ECM not only protects microbes from antimicrobial agents but also facilitates gene exchange among various species [27]. Therefore, the development of antimicrobial surfaces is important for reducing biofilm formation.

### 2.3. Main factors Affecting Cell-Surface Interactions

Interactions between microbes and the surrounding environment are governed by two factors: surface charge and degree of hydrophobicity. The cell wall of microbes is a vital component that physically separates the intracellular and extracellular environments, playing a vital role in cell physiology (particularly in nutrient exchange), signaling, and adhesion [28]. The surface charge is an important factor that affects the interactions between microbes and surfaces [15,29]. Most bacteria have a negatively charged surface; the charge originates primarily from ionized carboxyl and phosphate groups located on the bacterial surface [30]. For example, the cell wall of gram-positive bacteria comprises peptidoglycans embedded with teichoic acids, which are anionic cell surface polymers [16]. In addition, the outer membrane of gram-negative bacteria comprises phospholipids and lipopolysaccharides, which impart a net negative charge to the cell surface. [31] In fungi, mannoproteins exist on their cell walls and are linked to β-(1→6)-glucan through a remnant of a glycosylphosphatidylinositol anchor, inducing a negative charge on the fungal cell wall [32]. These microbes may form strong electrostatic interactions with the positively charged surfaces. The charge distribution on viral particles depends on the classification and arrangement of proteins within their structures, affecting the interactions between viruses and their surfaces [33,34]. In response to changes in the extracellular environment, the molecular composition of the microbial envelope changes, changing the surface charge density [35].

In addition, surface hydrophobicity affects microbial adhesion and detachment. For example, the high content of hydrophobic amino acid residues within bacterial pili enables bacteria to adhere to hydrophobic surfaces [36].Furthermore, increasing the length of the hydrophobic mycolic acid chain on the fungal surface improves the adhesion of fungi (such as *C. albicans*) to hydrophobic surfaces [37].

### 2.4. Surface-Associated Motility

Recent studies have indicated that bacterial motility is proportional to bactericidal efficiency [38]. Motility is considered one of the most vital behaviors of living organisms, allowing them to move toward their nutrient resources and distribute their progeny [39]. In nature, bacteria attach to biotic or abiotic surfaces. Various mechanisms of cell motility on surfaces can be observed in nature, including swarming, twitching, and gliding. Swarming is a phenomenon that describes the collection of locomotives by several organisms, such as bacteria, insects, and fish [40,41]. Swarming motility in bacteria is detected when flagellated bacteria grow on solid and wet surfaces [42,43]. On wet and nutrient-rich solid surfaces, vegetative bacterial cells differentiate into a new type called the swarmer phenotype, exhibiting hyperflagellation and an increase in cell length [44,45,46]. Twitching motility is the most common form of surface-associated motility, which is mediated by slender filaments termed “type IV pili” [47,48,49]. Gliding motility is defined as smooth bacterial movement over surfaces without the aid of flagella or pili, such as in myxobacteria, cyanobacteria, and flavobacteria [50]. Other types of motilities on antimicrobial surfaces have not been explored recently; however, the mobility of microbes can be an important factor in the design of antimicrobial surfaces.

## 3. Microbicidal Surface

### 3.1. Mechano-Bactericidal Surface

The concept of mechano-bactericidal surfaces was inspired by natural materials that possess unique mechano-bactericidal properties. For example, the *P. claripennis* cicada wing was first identified to possess a bactericidal function. The nanopillar array, 200 nm high with a cap diameter of 60 nm, a base diameter of 100 nm, and a pitch of approximately ~170 nm between the pillars, can penetrate *P. aeruginosa* that adheres to it, causing the bacterium to lose activity within a few minutes [51,52,53]. Furthermore, the surface remained bactericidal when the wing was coated with a 10 nm gold film [2]. Hasan reported that gram-negative bacteria such as *B. catarrhalis*, *E. coli*, *P. aeruginosa*, and *P. fluorescens* were killed by these nanostructures. However, gram-positive bacteria, such as Bacillus subtilis, *P. maritimus,* and *P. fluorescens,* with thicker cell walls remain active [53]. Kelleher reported that cicada wings with smaller nanopillar diameters and pitches (*M. intermedia* and *C. aguila*) have better bactericidal effects than *A. spectabile* [52]. Other natural surfaces, including dragonfly wings [54,55,56,57], damselfly wings [58], and gecko skin [59,60], were also determined to possess mechano-bactericidal activity. Therefore, these natural surfaces can be used as templates for the development of artificial antimicrobial agents.

In addition, various biocompatible materials have been explored for the development of artificial mechano-bactericidal surfaces. For example, a nanopillar array with a high aspect ratio was fabricated by reactive ion etching of silicon dioxide substrates (black silicon), which was inspired by the structure of dragonfly wings, with a nanopillar diameter of 20–80 nm and a height of 500 nm, which could eliminate gram-negative bacteria, gram-positive bacteria, and endospores [61]. By further integrating black silicon with microfluidic devices, *E. coli* was effectively destroyed in water [62]. Similarly, a mechano-bactericidal surface developed from the chemical etching of an aluminum alloy 6063 (AA6063) substrate was effective against both gram-positive (*S. aureus*) and gram-negative bacteria (*P. aeruginosa*) [63]. High-aspect-ratio carbon nanotubes and graphene have been reported to possess mechano-bactericidal effects [64]. Similar approaches to producing bactericidal surfaces, such as plasma etching (for silicon substrates) [65,66], hydrothermal etching (for titania, TiO_2_) [38], electrochemical etching (for stainless steel) [67], laser treatment (for metals) [68], and nanoimprint lithography (for polymers) [69,70], have also been reported recently (Table 1). Among these surfaces, significant variations were observed in the bactericidal performance. The surfaces comprising black silicon exhibited 99% bactericidal activity against gram-positive bacteria (*Staphylococcus aureus* cells) [65]. The surfaces comprising gold [71], zeolitic imidazole frameworks [72], polymers [70], and carbon nanotubes [73] exhibited bactericidal activity that could eliminate 99, 99, 100, and 93% of the attached bacteria, respectively. Other surfaces that exhibit weak activity against gram-positive bacteria or only carry bactericidal effects against gram-negative bacteria should be further optimized to meet the demands of practical applications. These artificial mechano-bactericidal surface properties have been critically evaluated and summarized in Table 1.

### 3.2. Bactericidal Mechanisms of Mechano-Bactericidal Surface

Bactericidal mechanisms may involve surface, microbial, or biological interactions. However, surface factors and surface deformation-induced biological reactions are the major issues discussed in this review.

**Figure 2:**
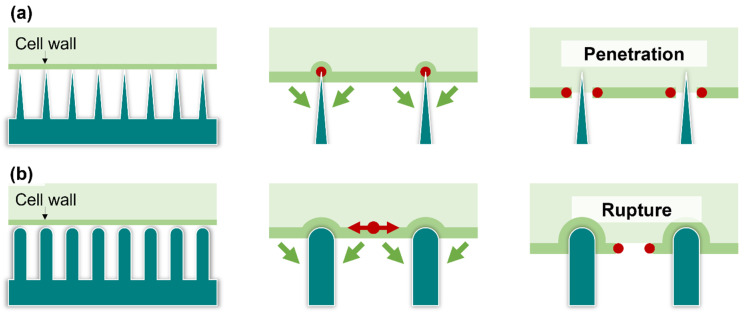
Mechanisms of bactericidal surfaces. (**a**) Penetration mechanisms shows that the cell wall of bacteria is penetrated by sharp structures of the bactericidal surface. (**b**) The stretching mechanism shows that the cell walls of bacteria may experience increased stretching tension and deformation as they land on nanostructures. The green arrows indicate the direction of cell wall movement on surface structures, and the red dots indicate the breakpoints of cell wall.

During the application of mechano-bactericidal effects to prevent bacterial surface adhesion and growth, several physical factors of the nanostructured surface may be manipulated to achieve a broad bactericidal effect. These include contact area (radius and shape), interspacing, array, aspect ratio, and rigidity. In a study by Ivanova et al. (2012), scanning electron microscopy and confocal laser scanning microscopy results indicated that *P. aeruginosa* undergoes unexpected deformation within minutes of contact with cicada wing surfaces and is eventually penetrated by the nanostructures [51]. Hence, penetration (Figure 2a) was initially proposed as the underlying mechanism responsible for the mechano-bactericidal effect of the cicada wings and mimicking nanostructures [51]. Furthermore, stretching (Figure 2b) was proposed as an alternative mechanism by Baulin et al. in 2013; they suggested that bacteria may experience increased stretching and deformation of their surface structures as they land and gradually coat the nanostructures [86].

The size of the contact area on the nanostructures is viral. According to the stretching mechanism hypothesis, a broad contact area may effectively stretch the suspended area of the cell membrane and cause cell rupture [87,88]. This was proven in a study by Mo et al. (2020), where the deformation of *E. coli* on a polyetheretherketone (PEEK) cone and pillar microstructure array was greater than that on a PEEK nanostructure array, probably owing to stronger cell adhesion [89]. Another experiment indicated the role of the additional adhesion area provided by the tapered slope of the sharp-tipped nanocone array in improving bactericidal efficiency [70,89]. Therefore, cell membrane rigidity was proposed to play an important role in stretching-based mechano-bactericidal effects; that is, more rigid cells require stronger interactions with the surface to stretch sufficiently for disruption [86].

The interspacing of micro/nanostructures also affects the bactericidal effects. First, a micro/nanostructure interspace that is less than the average size of the bacteria is desirable to avoid the settling of bacteria between the micro/nanostructures, thus losing the contact effect on the tip of the micro/nanostructures [90]. Computer simulations performed within this constraint further predicted that a larger interspace increases the stretching level of the bacterial cell membrane, providing a better sterilization efficiency [91]. However, an experimental anti-*S. aureus* study by Modaresifar et al. indicated that a nanostructure array with a 100 nm interspace exhibited the best sterilization efficiency using silicon nanoarrays, which were fabricated by electron beam-induced deposition [92]. This discrepancy was resolved by Yarlagadda, in which the simulation parameters were not consistent with a real experimental study. Therefore, Yarlagadda agreed with Modaresifar et al. that smaller interspaces increase sterilization efficiency [93].

Several studies have discussed the influence of the aspect ratio and rigidity of nanopillars on the sterilization effects. For structures that share the same width, those with a higher aspect ratio may have less structural rigidity (be softer) and may be deformed during the process of bacterial adhesion [94]. Such deformation and bundling are suggested to cause less cell membrane stress, thereby reducing the sterilization efficiency on the surface [95,96].

However, another study has shown that the wing of the cicada, *P. eyrie*, with a higher aspect ratio, demonstrated a higher sterilization efficiency than other cicada wings (*P. claripennis* and *A. curvicosta*) [94]. Moreover, Linklater supported previous observations with an extremely high aspect ratio of vertically aligned carbon nanotubes. Simultaneously, they observed that energy stored during the deformation of nanostructures may help increase the sterilization efficiency of nanostructure surfaces [83].

Notably, Ivanova indicated that deep-ultraviolet lithography-fabricated silicon nanopillar arrays possessed optimal *P. aeruginosa* and *S. aureus* killing efficiency at a certain nanopillar aspect ratio without pillar deformation [95]. These controversial results may be attributed to the intrinsic properties of different materials in micro/nanostructures.

As a result of investigating the effects of the nanostructure density on the bactericidal effects, Wu et al. indicated that there is an optimal range (40 pillars μm^−2^ with a surface roughness of 39.1 nm possesses the highest bactericidal efficiency) for eliminating *S. aureus* [70].

The hypothesis of both the penetration and stretching mechanisms was inferred from indirect experimental evidence, and rupturing of the cell membrane was thought to be the main cause of bacterial cell death. The penetration mechanism indicates that cell rupture occurs at the vertex in contact with the nanostructure, whereas the stretching mechanism indicates that cell rupture occurs in the suspended area between the nanopillars that is not in contact with the structure. The exudation of cell fluid in the bacteria also indirectly proves that the bacteria are killed during cell rupture [97]. In addition, several studies have reported that the cell wall of a bacterium repairs itself and maintains its integrity, vitality, and reproduction after being repeatedly penetrated by the sharp tip of an atomic force microscope [98].

The bactericidal activity of the nanostructured titanium dioxide surface against motile bacteria (such as *P. aeruginosa*, *E. coli*, and *B. subtilis*) was better than that against non-motile bacteria (such as *S. aureus*, *E. faecalis*, and *K. pneumonia*) [38], indicating that the mobility of bacteria and degree of membrane strength can affect the efficiency of bactericidal surfaces [38]. Bandara et al. (2017) employed a combination of advanced microscopy techniques to elucidate the bactericidal mechanism of dragonfly wings [54]. It has been suggested that rapid movement on such nanostructures may induce excessive shear force, which is caused by the strong adhesion between the nanopillars and EPS layer, damaging the bacterial cell membrane. However, this hypothesis does not explain the rupture of the non-motile bacteria. This mechanism was refuted because the bactericidal mechanism of nanostructures can be observed within a few minutes, whereas it takes hours to days for bacteria to secrete EPS [99,100]. Further studies on the interactions between bacteria and immobilized surfaces are required.

Recent studies have indicated that *S. aureus* and *E. coli* cells ruptured by titanium dioxide nanostructures may induce oxidative stress and cause cell death upon exposure [101,102,103]. These results suggest that stress-related cellular biochemical responses may be the dominant factor in microbial destruction after interaction with the designed nanostructures.

### 3.3. Virucidal Surfaces

The discovery of the microbial sterilizing effects of the surface of natural materials, such as metals and plant fibers, may be dated back to 2600 B.C.; an Egyptian medical text from that era describes the sterilization effects of copper surfaces on chest wounds and drinking water [104]. Since then, the use of these material surfaces (particularly copper and copper-related materials such as brass and bronze) for “contact killing”-based antimicrobial and decontamination purposes in hospital and public health settings has increased consistently [105,106]. Several surfaces fabricated by the direct coating of microbicidal metals in the form of ions, oxides, composites, and nanoparticles (NPs) or the integration of such metallic materials into the surface structure have been developed and used for contact-based microbial inactivation purposes, particularly for inactivating viruses [107]. For example, Cu-coated surfaces have been used to inactivate viruses such as influenza viruses, murine noroviruses, human immunodeficiency virus (HIV), and coronaviruses [108]. The zinc-coated surfaces are also effective in eradicating the measles virus, influenza, HIV, herpes simplex virus (HSV), rhinovirus (RV), hepatitis C virus, and coxsackievirus [109,110,111]. Recently, several metallic materials have been impregnated into commercial masks and fabrics for virus inactivation. For example, Borkow et al. (2010) impregnated copper oxide in N95 mask layers for the inactivation of the human influenza A virus (H1N1) and avian influenza virus (H9N2) [112], whereas Balagna et al. (2021) sputtered nanocluster/silica composites on masks to inactivate SARS-CoV-2 [113].

Several metal NPs have been reported to inactivate viruses upon contact and may be used as virucidal coatings on surfaces. For example, silver NPs are effective in inactivating various viruses, including HIV-1, respiratory syncytial virus (RSV), hepatitis B virus, HSV type 1, influenza virus, and monkeypox virus [114,115]. Recently, photothermic NPs have been explored for surface virucidal purposes [116]. For example, Lin et al. (2021) developed a graphene nanosheet-embedded carbon film-based photosterile mask, whereby most viruses were inactivated by the photothermal conversion effect on the mask surface, rendering the mask reusable [117]. In addition to masks, similar concepts can be used to create antiviral surfaces on other fabrics [114,118,119] or solid surfaces.

In addition to contact-based virucidal metal-coated surfaces, several surfaces lined with specifically coordinated atoms or step-edged nanostructures were determined to be capable of effectively binding and inactivating biomolecules such as proteins and viruses [120,121,122,123]. Hassan et al. (2020) produced a rough nanostructure in the form of randomly grouped parallel ridges on the surface of aluminum alloy 6030 (AA6030) by wet etching [85]. These surface nanostructures were later observed to inactivate SARS-CoV-2 within 6 h post-contact [85]. In addition, they have been proven to be effective in eradicating RSV and RV [63]. Although the antiviral mechanism of such nanostructured-etched surfaces is unclear, it provides an opportunity to discover a new effective antiviral structure with a similar nature. Several studies have revealed that viruses have shorter half-lives on porous materials than on non-porous materials [9,10,124,125,126]. Lai et al., who measured the half-lives of SARS-CoV-1 on porous and non-porous materials, determined that the viruses survived less than 1 d on porous paper, 5 min to 1 day on porous cotton, 5 day on non-porous glass, and 4–5 day on plastics [127]. In addition, Chin et al. (2020), who measured the half-life of SARS-CoV-2 on porous and non-porous materials, discovered that SARS-CoV-2 survived for approximately 3 h on porous materials such as thin paper and 7 day on non-porous materials such as plastics [9]. Hosseini et al. (2022) compared the antiviral effects of the same materials with different porosities [128]. The results indicated that the effects are related to surface water absorption, drying time, or viruses being trapped in the structures [128,129,130]. In contrast to the pillar structure required for the mechano-bactericidal surface, a porous structural morphology may be an important basis in designing antiviral surfaces and should be further explored.

## 4. Anti-Biofouling Surface

### 4.1. Superhydrophobic Surface

**Figure 3:**
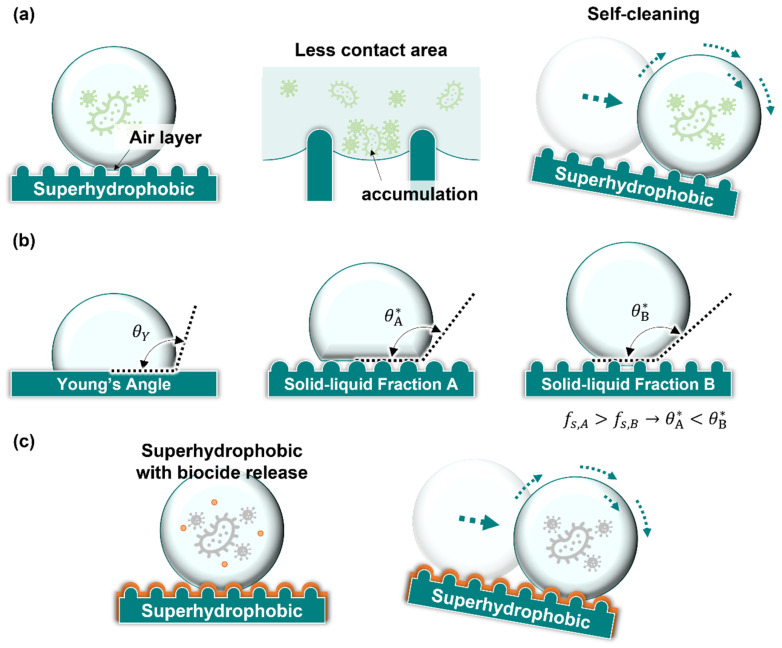
Antimicrobial mechanism of Cassie droplet on superhydrophobic surfaces. (**a**) Anti-biofouling mechanism of the Cassie droplet (compared with the Wenzel droplet) provides a smaller contact area between the liquid and the surface, allowing microbes to gather at the liquid-vapor interface between structures. At the same time, the cleaning characteristic of droplets takes the microbes off the surface. (**b**) Cassie droplet shows that the smaller the solid-liquid fraction, the greater the apparent contact angle is. (**c**) Superhydrophobic surfaces with biocide release kill microbes and bring the microbes off the surface. The green arrows indicate the direction of droplet movement and rotation on the superhydrophobic surface.

Lotus leaves possess a superhydrophobic surface that prevents dust and microbial retention. This ‘lotus effect’ can be used to develop a superhydrophobic coating surface that reduces bacterial surface adhesion [131,132]. Furthermore, the structure or roughness of the surface affects the macroscopic hydrophobic/hydrophilic properties of the surface and thus the microbial adhesion [133]. The droplets on the structural surface can be divided into two different states: (1) the Wenzel state, in which the droplet is immersed in the surface structures, and (2) the Cassie state, in which the droplet is suspended on the structure (Figure 3a). Further, the droplets in the Wenzel state (Wenzel droplets) often occur in rough, hydrophilic materials. Compared with droplets on a flat surface, Wenzel droplets immersed in the surface structures cause a lower contact angle and a higher hysteresis angle, which are not conducive to microbial removal on surfaces. Moreover, the structure and roughness induce bacterial adhesion, and smooth surfaces are negatively impacted during this process [134]. In contrast, a droplet in the Cassie state (Cassie droplet) is suspended in the structure, and there is an air layer between the droplet and surface. This provides a smaller contact area between the liquid and surface, and microbes can gather at the liquid-vapor interface between the structures (Figure 3a). Moreover, Cassie droplets have a high contact angle and a low hysteresis angle, making it easy for the droplet to move on the surface under the action of an external force (such as gravity or wind force). The moved droplet can further remove microbes from the surface; this phenomenon is termed ‘self-cleaning.’ Cassie droplets can be described by the Cassie-Baxter equation, as follows:

cos θ* = *fs* (cos *θ^Y^* + 1) − 1 (1)

θ* denotes the apparent contact angle, and *θ^Y^* the Young’s contact angle, which is the contact angle of the material itself. fs denotes the solid-liquid fraction, which is the ratio of the liquid and solids on the surface. Typically, the smaller the contact area of the solids and liquids (*fs*), the greater the apparent contact angle (θ*), and the lower the hysteresis angle. The superhydrophobic surfaces can be produced by the surface coating or Young’s contact angle of the material and a properly designed solid–liquid contact area (fs).

A surface may be identified as superhydrophobic when the apparent contact angle of a water droplet with the surface is >150° and the hysteresis angle is <10° [133]. The droplets easily roll on superhydrophobic surfaces, facilitating the removal of surface microbes [133].

The sterilization materials (such as metals, metal oxides, quaternary ammonium compounds, and *N*-halamine compounds) are often incorporated into superhydrophobic surfaces during surface fabrication to provide surfaces with both bactericidal and surface microbe removal functions [135], (Figure 3b).

**Figure 4:**
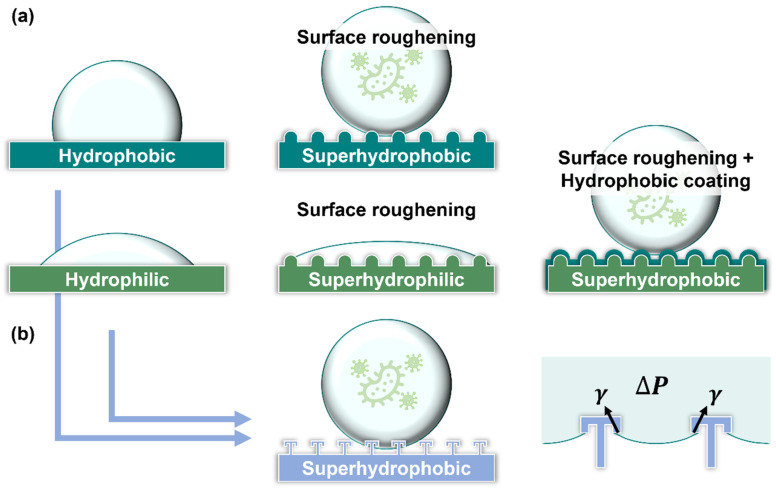
Approaches to obtaining superhydrophobic surfaces (**a**) Superhydrophobic methods, such as roughening hydrophobic surface or hydrophobic coating to a rough surface. (**b**) DRT structures provide upward surface tension for droplet suspension even on highly wetted materials, which have mechano-superhydrophobic and anti-biofouling characteristics.

High surface roughness and low surface energy are essential for the formation of superhydrophobic surfaces [133]. Therefore, two approaches are typically employed during the production of superhydrophobic surfaces: (1) coating low-surface-energy materials on rough surfaces or (2) roughening the surface of low-surface-energy materials (Figure 4a) [136,137].

The first approach is typically applied to cotton fabrics (which possess rough surfaces) to produce antimicrobial superhydrophobic surfaces. For example, Gao et al. (2021) applied Ag NPs and hydrophobic polydimethylsiloxane (PDMS) or polyimide (PI) coatings onto the cotton fabric [138], whereas Liu et al. (2021) applied electrostatic adsorption of Ag/AgCl, PDMS, and PI to the cotton fabric [139] to confer bactericidal and superhydrophobic properties to the fabric. However, Wu et al. produced a superhydrophobic fabric that contained silver stearate with UV-curable waterborne coatings, Ag NPs, and stearic acid [140], whereas Shaban et al. (2018) produced ZnO NP gel-coated bactericidal superhydrophobic fabrics [141]. Lai et al. (2021) immersed polyethylene terephthalate fabrics in a solution containing ZnO NPs and PDMS followed by low-pressure Ar plasma treatment to produce ZnO PDMS fabrics that possess sterilizing and superhydrophobic properties [142]. Suryaprabha and Sethuraman (2017) plated copper on cotton fabrics (Cu-coated cotton) and hydrophobized it with stearic acid to achieve bactericidal and super-hydrophobic properties [143]. Raeisi et al. (2021) soaked cotton fabric in a chitosan solution containing hydrophobic fumes of titanium dioxide NPs, which made the fabric bactericidal and superhydrophobic [144]. Aslanidou and Karapanagiotis (2018) sprayed a solution containing alkoxy silanes, organic fluoropolymers, silane quaternary ammonium salts, and silica NPs on silk to endow it with bactericidal and superhydrophobic characteristics [145]. Song et al. (2019) fabricated bactericidal superhydrophobic coatings with quaternary ammonium salt-modified nanosilica to produce fabrics with both bactericidal and superhydrophobic properties [146].

In addition to coating specific materials on rough surfaces, several other methods have been used to produce rough surfaces on low-energy materials. Agbe et al. (2020) fabricated an Ag-polymethylhydrosiloxane coating on anodized aluminum, which provided surface roughness to achieve bactericidal and superhydrophobic properties [147]. Spasova et al. (2017) produced polyvinylidene fluoride (PVDF) and PVDF-co-hexafluoropropylene superhydrophobic nanofibers containing ZnO using a one-pot electrospinning technique that exhibited both superhydrophobic and bactericidal properties [148]. Wang et al. (2021) produced superhydrophobic coatings using an electrodeposition-grafting modification method on indium tin oxide, which was also bactericidal [149]. Ren et al. (2018) spray-coated hydrophobic silica sol and CuO NPs onto glass to produce a highly transparent superhydrophobic surface with bactericidal effects [150]. Subhadarshini et al. (2019) fabricated Cu_2_O nanopetals on Cu foil by electrochemical deposition [151]. These surfaces exhibit both superhydrophobic and bactericidal properties. Duan et al. (2020) fabricated wood with bactericidal and superhydrophobic functions by the self-polymerization of dopamine, chemical deposition of Cu NPs, and hydrophobic modification of fluorosilane [152]. Agbe et al. (2020) chemically etched aluminum alloy 6061 (AA6061) to create rough and modified low-surface-energy octyltriethoxysilane molecules to produce superhydrophobic coatings [153]. Bartlet et al. (2018) fabricated titania nanotube arrays on titanium using an anodizing process and chemical vapor deposition of (heptadecafluoro-1,1,2,2-tetrahydrodecyl)trichlorosilane for surface modification [154]. The surfaces of these nanotubes are superhydrophobic, decreasing the adhesion of proteins and bacteria. These superhydrophobic surface properties have been critically evaluated and summarized in Table 2.

The superhydrophobic surfaces that do not require the coating of additional materials for antimicrobial purposes have significant potential for industrial applications owing to their long service lives and lower structural limitations (owing to the absence of a coating). A notable example is the double reentrant topology (DRT) surfaces that possess anti-biofouling properties and antibacterial adhesion effects (Lee et al., 2022) [155]. A DRT is a structure with a negative sidewall angle, such as in mushrooms or umbrellas (Figure 4b). Unlike other superhydrophobic surfaces, DRT does not require the use of low-surface-energy materials to achieve superhydrophobicity, relying on its special structure [156]. In a study by Lee et al. (2022), DRT surfaces were observed to remain superhydrophobic and exhibit low microbial adhesion behaviors, even when hydrophilic and microbe adhesive materials (SiO_2_) were used as contact materials [155]. The DRT surfaces can be considered mechanical anti-biofouling surfaces and may serve as an example that inspires the development of novel antibacterial surfaces.

### 4.2. Slippery Surface

The slippery liquid-infused porous surfaces (SLIPS) are surfaces with smooth, continuous liquid layers fabricated under the inspiration of the inner wall surfaces of the pitcher of the nepenthes [157,158]. The Nepenthes pitcher wall surfaces exhibited high water absorption. This creates a liquid layer on the wall in humid environments, causing insects to slide toward the bottom of the pitcher for digestion [159,160]. The SLIPSs employ similar concepts to minimize microbial adhesion on the surface.

**Figure 5:**
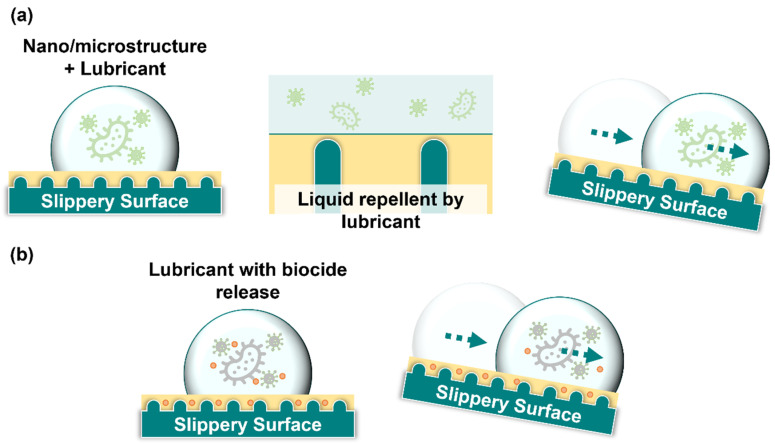
Antimicrobial mechanisms of slippery surfaces. (**a**) The anchored lubricant layer forms a slippery surface that prevents microbes from contacting and colonizing surfaces, hence preventing microbial adhesion. (**b**) A slippery surface can be preloaded with biocides on the structure substrate or in the lubricant to kill microbes. The green arrows indicate the direction of droplet movement on the slippery surface.

SLIPS are fabricated by anchoring a layer of lubrication liquid film on a surface with the assistance of surface micro/nanostructures (Figure 5a). The key steps in constructing the SLIPS include surface texturing, low-surface-energy modification, and injection of lubrication. Three standards should be followed when designing SLIPS: (1) lubrication should stably adhere to the micro/nanostructured surface texture; (2) the lubricating liquid must wet the micro/nanostructured surface texture more readily than the repelled liquid; and (3) the lubricating and repelled liquids must be immiscible and incompatible [157].

Several technologies have been used to develop micro/nanostructured surface textures for SLIPS. These include anodization [161], wire electrical discharge machining [162], electrochemical texturing [163], and thermally induced phase separation [164]. In comparison with superhydrophobic surfaces, SLIPS possess excellent liquid repellency, corrosion resistance, anti-icing properties, and enhanced durability [165]. In addition, the liquid layer of SLIPS can effectively prevent microbes from contacting and colonizing the surface, preventing microbial adhesion.

Several SLIPS have been developed and applied in industrial settings. For example, Epstein et al. (2012) fabricated a micro/nanostructural matrix on silicon wafers using the Bosch process, hydrophobized the surface with heptadecafluoro-1,1,2,2-tetrahydrodecyl trichlorosilane gas, and injected lubrication liquid to form SLIPS with excellent antibacterial-adhesion characteristics [166]. In addition, Epstein et al. (2012) indicated that a commercially available porous Teflon film can be used as a substrate to produce SLIPS by similar techniques without additional modification [166]. The lubrication liquid of the SLIPS used by Epstein et al. (2012) was a perfluorinated liquid, including perfluoropolyethers (PFPE), perfluorotripentylamine, and perfluorodecalin, preventing the adhesion of various bacteria for 7 day [166].

Li et al. (2013) produced a macroporous poly(butyl methacrylate-co-ethylene dimethacrylate) surface and injected PFPE liquid to form SLIPS with anti-biofouling and antibacterial adhesion properties [167]. However, Leslie et al. (2014) tethered perfluorocarbon on a surface with covalently bound perfluorodecalin and injected it to form medical-grade SLIPS [168]. This surface effectively prevents the attachment of P. aeruginosa and the formation of biofilms [168]. Howell et al. (2014) produced self-replenishing vascularized fouling-release surfaces with PDMS [169]. This surface was turned into a SLIPS by soaking PDMS in silicone oil, and the SLIPS properties were maintained by dedicated channels that consistently replenished silicone oil to the surface [169]. This study determined that a vascular network with lubricating fluid filling can possess the ability to increase the antifouling lifespan of the surface.

Although the anti-biofouling and antibacterial adhesion properties of SLIPS have been established, they do not possess any bactericidal effect. Recent studies have leaned toward the development of SLIPS with bactericidal and anti-biofouling capabilities (Figure 5b). Manna et al. (2016) deposited porous polyethyleneimine (PEI)/poly(2-vinyl-4, 4-dimethyl azlactone) (PVDMA) multilayers on glass substrates by repeatedly soaking them in PEI solution and amine-reactive PVDMA solution. The SLIPS were obtained by coating a porous structural surface functionalized with a decylamine solution with silicon oil [170]. This study indicated that loading broad-spectrum antimicrobial biocides, such as triclosan, onto porous structural layers or mixing with injected silicone oil can produce bactericidal SLIPS [170]. Kratochvil et al. (2016) loaded small-molecule quorum-sensing inhibitors (QSI) onto PEI/PVDMA structural layers and obtained SLIPS by injecting silicone oil [171]. The QSI was shown to be gradually released into the surrounding medium to provide an antibacterial effect, with the rate of release controlled by the structure [171].

Lee et al. (2019) produced poly(pentafluorophenyl acrylate), a porous surface (termed PAR film), by a selective removal method [172]. The PAR film underwent surface modification by soaking in an aqueous solution of dopamine hydrochloride, PDMS/amine-PDMS/hexane solution, and AgNO_3_ solution [172]. The modified PAR film was then injected with silicon oil to form a SLIPS surface with bactericidal and anti-biofouling properties [172]. This surface exhibits excellent antibacterial effects against *E. coli* dispersed in water and air [172].

Wylie et al. (2020) obtained SLIPS by injecting phosphonium ionic liquids (PILs) into poly(vinyl chloride) surfaces. The PILs possess antibacterial properties, providing a surface with dual functions of bactericidal and antibacterial adhesion [173]. Hao et al. (2022) fabricated a dagger-shaped, oriented zeolitic imidazolate framework layer and injected a fluorinated lubricant oil to create SLIPS [174]. This surface was designed to provide antimicrobial adhesion by SLIPS mechanisms at the initial phase and a mechano-bactericidal effect by the dagger-shaped structure at a later stage when the lubricant layer is consumed [174].

Zang et al. (2022) used poly(ethylene glycol) as a sacrificial template to form a porous polystyrene structure using microphase separation technology. After covalent modification with 3-(trimethoxysilyl)propyl dimethyl undecyl ammonium chloride (QAC-silane) and the injection of silicon oil, SLIPS was produced [175]. This surface is repellent to various bacteria, and after the lubricating fluid is depleted, the exposure of the QAC allows the surface to remain bactericidal.

Therefore, microbial adhesion to the surface may be influenced by surface structures and materials [175]. These slippery surface properties have been critically evaluated and summarized in Table 3.

## 5. Summary and Perspectives

Microbes such as bacteria, fungi, and viruses are typically found on various surfaces. Their high adaptability to survival is often accompanied by their ability to adhere to various surfaces. Several medical problems have been caused by the contamination of surfaces with infectious pathogens, which has led to an increasing number of infected populations worldwide. The different chemical substances are used as antiseptics or disinfectants to prevent or reduce infections. However, chemical abuse may create new pathogens that are highly resistant to antiseptics and disinfectants and are known to develop antimicrobial resistance. Therefore, physical approaches to kill microbes and prevent their attachment to surfaces have been developed.

Cicada wings have inspired scientists to fabricate a surface topology with a mechano-bactericidal effect; for example, fabricated nanopillars of silicon dioxide substrates and TiO_2_ are used to kill both gram-negative and gram-positive bacteria. Furthermore, we discussed the mechanical penetration or rupture of microbial cell mechanisms or tensile stress-induced reactive oxygen species responses.

The nanopillar arrays with high aspect ratios and sharp nanostructures are more effective bactericidal structures. The structures for different array parameters were also discussed. However, the effects of mechanically bactericidal surfaces were notable. Due to the fact that this type of surface is suitable for use on a variety of materials, it has the potential to be used in various applications. However, these structures are not antiviral in nature. Owing to the significant differences in size between viruses and bacteria, the structural size of the current bactericidal surfaces cannot stretch or penetrate viruses. In addition, shrinking the structural size may be a method for achieving a virucidal surface in the future, and the mechanism of mechano-bactericidal surfaces may provide a mechanism for a mechano-virucidal surface. Moreover, there was a significant difference between the half-life of the virus on non-porous and porous materials, which may provide another explanation for the design of the mechano-virucidal surface.

The superhydrophobic surfaces and SLIPS can prevent the adhesion of various microbes. Notably, the use of a double-reflex structure can transform various materials into superhydrophobic surfaces and has been proven to be both bactericidal and antiviral. Similar to mechano-bactericidal surfaces, surfaces that are less affected by materials have potential for various applications. This provided new evidence for the development of such surfaces.

Inspired by nepenthes, the SLIPS is a promising research area that depends on the fabrication of surfaces with layers of lubrication to prevent the attachment of microbes to surfaces. In addition, due to the fact that the SLIPS do not have direct contact with solids on their surfaces, they possess excellent anti-biofouling characteristics. Currently, the depletion or replenishment of lubricants on SLIPS is a major problem that directly affects the timeliness of surface functionality. Further, studies tend to add a bactericidal agent to the structure or lubricant of SLIPS antimicrobial surfaces to make the surfaces not only anti-biofouling but also able to kill microbes.

The self-cleaning and antimicrobial surface developments are highly desirable to reduce contact transmission. This review summarizes the latest progress in structure-related mechanisms of antimicrobial surfaces. Although it is tempting to believe that antimicrobial surfaces can be achieved using only structural mechanisms, such as mechano-bactericidal surfaces and DRT-based superhydrophobic surfaces, it is not the purpose of this review to advocate for the development of such structural surfaces. This article aims to provide new insights into the development of antimicrobial surfaces by discussing the role of current structures on antimicrobial surfaces.

## Figures and Tables

**Table 1:** Summary of artificial mechano-bactericidal Surfaces.

Material	Substrate	Fabrication	Structure Type	Structure Dimensions (nm)			Microbes (Bactericidal Activity)			Notes and/or Potential Applications	Refs
				Diameter	Pitch	Height	Gram-Negative	Gram-Positive	Others		
Black Silicon (Si/SiO2)	Silicon	Reactive ion beam etching (RIE)	Nanopillar	20–80	NI *	500	*P. aeruginosa* (~50% after 3 h)	*B. subtilis* (~15% after 3 h), *S. aureus* (~50% after 3 h)	NT *	First artificial mechano-bactericidal surfaces device	[61]
				62	62	280	*P. aeruginosa* (89%)	*S. aureus* (85%)	NT	High bactericidal activity	[74]
			Nanograss	10–20	NI	4000	*E. coli* (83% after 3 h)	*S. aureus* (86% after 3 h)	NT	May cause mammalian cells injury or death Not suitable for biomedical implants	[75]
		Plasma etching	Nanopillar	150–200	100–250	NI	*E. coli* (~95% after 3 h, ~99% after 24 h)	*S. aureus* (~63% after 3 h, ~99% after 24 h)	*B. cereus* (~30% after 3 h, ~99% after 24 h)	Effective against spore-forming bacteria	[65]
Titanium (Ti)	Titanium	Glancing angle sputter deposition (GLAD)	Nanocolumn	NI	158	478	*E. coli* (60%)	*S. aureus* (N/A *)	NT	Not affect tissue-like cells (hMSCs) and leukocytes (PBMCs)	[76]
Titania (TiO_2_)	Titanium	Hydrothermal process	Nanowire	100	NI	3000	*P. aeruginosa* (>60%), *E. coli* (>60%), *K. pneumonia* (<5%)	*S. aureus* (<5%,) *B. subtilis* (>60%), *E. faecalis* (<5%)	NT	Enhances the proliferation of mammalian cell (such as human osteoblast-like cells). Suitable for biomedical applications	[38]
			Nanopattern array	40	NI	NI	*P. aeruginosa* (50%)	*S. aureus* (20%)	NT	Enhances the proliferation of primary human fibroblasts (PHF). Suitable for biomedical applications	[77]
			Nanospike	10–30	2000	2000	NT	*S. aureus* (15%)	NT	Combination of nanostructure and antibiotic coating May be suitable for biomedical implants	[78]
			Nanospears	50	3000–5000	4000	NT	*S. epidermidis* (47%)	NT	Retard biofilm formation Suitable for biomedical implants	[79]
Black Titanium (Ti/TiO_2_ /Cl)	Titanium	Chlorine-based reactive ion etching (RIE)	Nanopillar	80	random	1000	*E. coli* (95% after 4 h), *P. aeruginosa* (92% after 4 h)	*S. aureus* (~22% after 4 h, ~76% after 24 h), *M. smegmatis* (~92% after 4 h)	NT	Broad-spectrum antibiotic activity Compatible for human mesenchymal stem cells. Suitable for biomedical implants	[80]
Gold (Au/W)	Silicon	Sputtering W and Al film, Al film anodization, Au electrodeposition	Nanopillar	50	NI	100	NT	*S. aureus* (~99%)	NT	Cost-efficient Fabrication Scalable fabrication process	[71]
		Sputtering W and Al film, Al film anodization, W plasma etching, Au electrodeposition	Nanoring	200	NI	100					
Polymer	Glass	Nanoporous template molding, ormostamp solution, UV curing	Nanopillar	80	130	200	NT	*S. aureus* (23%)	NT	Optimum NP density: ∼40 pillars μm^−2^	[70]
				80	170	400		*S. aureus* (100%)			
				80	170	200		*S. aureus* (98%)			
				80	300	300		*S. aureus* (26%)			
PMMA	PMMA	Nanoimprint lithography (NIL)	Nanopillar	70	170	210	NT	NT	*A. fumigatus* *F. oxysporum*	Inhibits the growth of filamentous fungi	[81]
				120	320	300					
				100	500	700					
PC	PC	Nanoporous anodic aluminum oxide (AAO) template-assisted hot embossing, wet etching	Nanopillar	<60	170	200	*E. coli* (98.4%)	NT	NT	Polymer nanostructure surfaces	[82]
Carbon nanotubes	Silicon	Chemical vapor deposition (CVD)	Vertically aligned nanotube forest	10	<10	20,000	*E. coli*, (N/A) *P. aeruginosa* (N/A)	*B. subtilis*, (45%) *S. epidermidis* (~90%)	NT	Plasma treatment may affect bactericidal activity	[73]
				10	<10	1000	*P. aeruginosa* (99%)	*S. aureus* (84%)	NT	Exceptionally high aspect ratio (100–3000) nanostructure Storage and release of mechanical energy	[83]
				10	<10	30,000		*S. aureus* (17%)			
Graphene	Glass	liquid-phase exfoliation procedure, vacuum filtration process	Nanoblade	5	NI	Horizontal length 79.7	*P. aeruginosa* (71.4%)	*S. aureus* (77.1%)	NT	Graphene sheets: different edge lengths and different angles of orientation Density of the edges is important for bactericidal activity	[84]
Zeolitic Imidazole framework (ZIF)	Multi-substrate compatible	Coating	Nanodagger	2000	<2000	1000	*E. coli* (~99%)	*S. aureus* (~99%)	*C. albicans* (~80%)	Dagger-shaped nanostructure Positively charged surfaces enhances bactericidal activity (bacterial cell adhesion)	[72]
Aluminum alloy (AA)	Aluminum alloy 6063	Sodium hydroxide based wet etching	Nanopillar	23	161	NI	*P. aeruginosa* (92% after 3 h),	*S. aureus* (87% after 3 h)	Respiratory Syncytial Virus (RSV), Rhinovirus (RV) (3–4 log reduction)	With both antibacterial and antiviral activity	[63]
							NT	NT	SARS-CoV-2 (2.5 log reduction)		[85]

*: NI: No information from the cited references or not applicable; NT: Not tested. N/A: Not applicable or very low activity.

**Table 2:** Summary of superhydrophobic surfaces.

Substrate	Structure and Material	Fabrication	Microbes		Note and/or Potential Applications	Refs
			Gram-Negative	Gram-Positive		
Cotton fabric	PDMS/AgNPs/PDA PI/AgNPs/PDA	PDA modification, and immersing	*E. coli*	*S. aureus*	Good anti-corrosion properties and adequate durability Suitable as flexible wearable materials	[138]
	PDMS/Ag/AgCl	PDA modification, electrostatic adsorption, and immersing	*E. coli*	*S. aureus*	Great tolerance and resistance to broad pH (1–13) and various organic solvents Outstanding mechanical durability	[139]
	UV-curable waterborne coatings, the silver nanoparticles, and the stearic acid	Electric spraying, UV curing, immersing, and stearic acid modification	*E. coli*	*S. aureus*	Good resistance to the water and acid medium Silver nanoparticle: bactericidal agent	[140]
	ZnO NPs	Sol-gel method, and spin coating	*K. pneumonia*, *P. aeruginosa*, *E. coli*, and *S. typhimurium*	*S. aureus*, *B. subtilis*, *E. faecalis*, and *B. cereus*	Low fabrication cost, and large-scale fabrication availability ZnO NPs: a potent bactericidal agent with	[141]
	Cu/stearic acid	Chemical reduction of copper acetate and stearic acid immersing	*E. coli*	*S. aureus*	Cost-effective fabrication Good durability Cu: a bactericidal agent	[143]
	chitosan/TiO_2_ nanocomposites	Immersing	*E. coli*	*S. aureus*	Promising UV-protecting properties	[144]
	HDTMS, EPDDAC, and SiO_2_ NPs	Immersing	*E. coli*	*S. aureus*	Good washability	[146]
PET fabric	PDMS/ZnO NPs	Immersing, and Ar plasma treatment	*E. coli*	*S. aureus*	Good durability. ZnO NPs: a potent bactericidal agent	[142]
Silk	alkoxy silanes, organic fluoropolymer, silane quaternary ammonium salt, and silica nanoparticles	Spray	NI * (Microorganisms)		Good durability Potential application: protection of textiles of the cultural heritage	[145]
Anodized Al oxide (AAO)	Ag/PMHS nanocomposites	Dip-coating deposition	*P. aeruginosa*, and *E. coli*	*S. aureus*	Good stability of immersion in saline water Excellent scratch resistance and strong adhesion property	[147]
nanofibrous PVDF and PVDF-HFP mats	PVDF, PVDF-HFP, and nanosized zinc oxide with a silanized surface	One-pot electrospinning technique	*E. coli*	*S. aureus*	ZnO NPs: a potent bactericidal agent	[148]
ITO Glass	PDMS/ZnO	Electrodeposition-grafting modification method	*E. coli*	NT *	Good stability under UV irradiation and acid-base environment Excellent photocatalytic performance	[149]
Glass	hydrophobic silica sol, and CuO NPs	Oxygen plasma treatment, and spray-coating	*E. coli*	NT	Highly transparent Potential applications: biosensors, microfluidics, bio-optical devices, household facilities, lab-on-chips, and touchscreen devices	[150]
Copper foil	Cu_2_O nano pedals	Electrochemical deposition (ECD) method	*E. coli*	*B. subtilis*	Simplistic, eco-friendly, economical, and scalable fabrication method Cu_2_O: a bactericidal agent	[151]
Wood	CuNPs	PDA modification, coating, and fluorosilane treatment	*E. coli*	*S. aureus*	Good stability in a variety of harsh conditions containing strong acid/alkali, high temperature aging, and mechanical abrasion Suitable for wood veneer. CuNPs: a bactericidal agent	[152]
Aluminum alloy	OTES, and QUATs	Chemical etching, and immersing	*P. aeruginosa*, and *E. coli*	*S. aureus*	Suitable applications: anti-biofouling healthcare consumables such as nose masks, bedsheets and medical scraps	[153]
Titanium sheets	(heptadecafluoro-1,1,2,2-tetrahydrodecyl) trichlorosilane, and titania nanotube	Anodization process, and chemical vapor deposition (CVD)	*P. aeruginosa*,	*S. aureus*	Against biofilm formation Suitable for implanted medical devices	[154]

*: NI: No information from the cited references or not applicable; NT: Not tested.

**Table 3:** Summary of slippery surfaces.

Substrate	Material	Lubricant	Fabrication	Microbes	Note and/or Potential Applications	Refs
Silicon	Si/SiO_2_/heptadecafluoro-1,1,2,2-tetrahydrodecyl trichlorosilane	Perfluoropolyether, perfluorotripentylamine, or perfluorodecalin	Bosch process, vapor coating	*S. aureus*, *P. aeruginosa*, *E. coli*	Stable in submerged, extreme pH, salinity, and UV environments	[166]
Commercially available porous Teflon film	NA		NA			
Porous BMA-EDMA	BMA-EDMA	PFPE liquid	Coating	*P. aeruginos*	This device may have different surface antibiofouling ability of the same bacterial strain from field or laboratory.	[167]
Perfluorocarbon	Tethered perfluorocarbon	Perfluorodecalin	Plasma modification	*P. aeruginos*	Medical-grade perfluorocarbon Prevent thrombotic occlusion and biofouling of medical devices	[168]
PDMS	PDMS	Silicon oil	Micro molding, and immersing	*S. aureus*, *E. coli*, *green microalgae*, *Botryococcus braunii*, *Chlamydomonas reinhardtii*, *Dunaliella salina*, and *Nannochloropsis oculata*	With lubricant self-replenishment function of this device	[169]
Glass	PEI/PVDMA multilayers, triclosan loading	Silicon oil	Repeatedly soaking	*C. albicans*	Antimicrobial agent (triclosan) releasing to kill non-adherent pathogens	[170]
Glass	PEI/ PVDMA multilayers, QSIs loading	Silicon oil	Submerged iteratively	*P. aeruginos*	Small-molecule quorum sensing inhibitors (QSIs) releasing to attenuate virulence phenotypes in non-adherent cell	[171]
PAR film	PPFPA porous surface AgNPs loading	Silicon oil	Selective removal method, soaking modification	*E. coil*	AgNPs releasing to attenuate virulence phenotypes in non-adherent cell	[172]
Roughened PVC	PVC	Phosphonium ionic liquids (PILs)	NI	*S. aureus*, and *P. aeruginos*	The PIL: a potent bactericidal agent	[173]
Silica wafers, silicone, and polyurethane films or catheters	Oriented zeolitic imidazolate framework-L (ZIF-L) layer	Fluorinated lubricant oil	seeding and secondary growth technique	*S. aureus*, and *P. aeruginos*	Dual functions: mechano-bactericidal activity and lubricant antibiofouling	[174]
Glass slide, PC plate, PET plate, PE film, and silicone catheter tube	PS porous surface	Silicon oil	Dip coating, plasma treatment	*S. aureus*	Simple, low-cost, fast, and multi-substrate available fabrication	[175]

NI: No information from the cited references or not applicable; NT: Not tested.
